# Fast-track recovery program after cardiac surgery in a teaching hospital: a quality improvement initiative

**DOI:** 10.1186/s13104-021-05620-w

**Published:** 2021-05-22

**Authors:** Patryck Lloyd-Donald, Wen-Shen Lee, James W. Hooper, Dong Kyu Lee, Alice Moore, Nikhil Chandra, Peter McCall, Siven Seevanayagam, George Matalanis, Stephen Warrillow, Laurence Weinberg

**Affiliations:** 1 Department of Anesthesia, Austin Health, Melbourne, VIC Australia; 2 Department of Anesthesiology and Pain Medicine, Guro Hospital, Korea University School of Medicine, Seoul, Korea; 3 Department of Cardiac Surgery, Austin Health, Melbourne, Australia; 4 Department of Surgery, The University of Melbourne, Austin Health, Melbourne, Australia

**Keywords:** Cardiac anesthesia, Fast-track anesthesia, Quality improvement, Perioperative flow, Resource allocation

## Abstract

**Objective:**

Fast-track cardiac anesthesia (FTCA) is a technique that may improve patient access to surgery and maximize workforce utilization. However, feasibility and factors impacting FTCA implementation remain poorly explored both locally and internationally. We describe the specific intraoperative and postoperative protocols for our FTCA program, assess protocol compliance and identify reasons for FTCA failure.

**Results:**

We tested the program in 16 patients undergoing elective cardiac surgery requiring cardiopulmonary bypass. There was 100% compliance with the FTCA protocols. Four (25%) patients successfully completed the FTCA protocol (extubated < 4 h postoperatively and discharged from the intensive care unit on the same operative day).

## Introduction

Cardiovascular disease is common, accounting for almost 14% of the total burden of disease in Australia, resulting in a significant consumption of healthcare resources and estimated 8.9% ($10.4 billion AUD) of total disease expenditure per annum [1]. Over 12,000 Australians receive cardiothoracic surgery each year. Whilst the reported median waiting times for cardiothoracic surgery have increased from 16 days in 2013/4 to 19 days in 2017/8, the median waiting times for Indigenous Australian undergoing elective cardiac artery bypass surgery is 11 days [2]. The demand for cardiac surgery is predicted to increase in future, necessitating improved utilization of healthcare resources [3].

Fast-track cardiac anesthesia (FTCA) offers an alternative to standard cardiac surgical care, with improved efficiencies that do not compromise safety or clinical outcomes [4]. FTCA has gained increased interest in response to resource constraints and increasing demand [5]. Further, FTCA offers both a clinical and health-administrative paradigm shift in the way care is delivered to cardiac surgery patients, offering shorter ventilation times, shorter intensive care unit (ICU) length of stay and associated cost savings [6].

Evidence from a major review into FTCA demonstrated no difference in adverse events between patients receiving FTCA and standard care [4]. Our organization provides care to an increasing number of cardiac surgical patients, with an increasing demand for intensive-care admissions annually. The development of an FTCA protocol offers a potential solution to this problem. As a result, our organization aimed to implement a dedicated intraoperative and postoperative FTCA protocol. The primary aim was to determine if our FTCA program was feasible.

## Main text

### Materials and methods

Our organization is a major tertiary hospital within Melbourne, Australia. The organization provides transplant, cardiothoracic and neurosurgical sub-specialty elective and emergency surgery. It offers a capacity of 400 acute in-patient beds and a 30-bed ICU. At the time of this study, our service provided 543 cardiac surgeries annually, of which 401 were elective, primary bypass or valve operations.

In accordance with international quality improvement standards, we implemented a plan-do-study-act (PDSA) quality improvement process for our cardiac surgical patients [7]. We assembled a multi-disciplinary expert working group, who met weekly for a 3-month period to strategize, execute and evaluate a FTCA program. The specialist working group consisted of cardiac surgeons, anesthesiologists, intensivists, anesthetic nursing staff, intensive care nursing staff and hospital administrators. The working group subsequently agreed on an operational definition of FTCA being extubation within 4 h postoperatively *and* successful patient discharge to the ward from ICU within 24 h postoperatively [8]. We received organizational support from the hospital executive to implement the FTCA program, and organizational funding to facilitate treatment for one FTCA patient per week for a 16-week period. We then completed this quality improvement project to investigate the feasibility of the FTCA program.

Our working group developed a defined perioperative protocol for the intraoperative anesthetic management of each FTCA patient in order to standardize the anesthetic care, enhance clinician familiarity, and promote early-extubation. Compared to standard care, the anesthetic protocol focused on minimizing the use of sedating pre-medications, encouraging the use of shorter acting intraoperative opiates and adherence to a standardized postoperative care pathway (Table 1).

**Table 1:** Technique for fast-track cardiac anesthesia compared to standard care protocol

Standard care	Fast-track protocol
Preoperative medication	Preoperative medication
Oral benzodiazepine (temazepam 10 mg or diazepam 10 mg) Intramuscular morphine 10–20 mg, 1 h before surgery	Nil premedication

Insertion of vascular access (conscious sedation)	Insertion of vascular access (conscious sedation)
At discretion of treating anesthesiologist: midazolam (3–5 mg IV) or diazepine, morphine (5–10 mg) or oxycodome (5–10 mg)	Midazolam (1–3 mg IV), fentanyl (50–100 ug)

Induction of anesthesia	Induction of anesthesia
Opioid at discretion of treating anesthesiologist	Fentanyl (3–5 ug/kg IV) or alfentanil (30–50 ug/kg IV)
Neuromuscular blocking agent selected at discretion of treating anesthesiologist	Rocuronium (1 mg/kg) or vecuronium 0.1 mg/kg for neuromuscular blockade

Maintenance of anesthesia/analgesia	Maintenance of anesthesia/analgesia
Intraoperative opioids, benzodiazepines, volatile agents, and total intravenous anesthesia is at the discretion of the treating anesthesiologist	Remifentanil: (0.1–0.3 ug/kg/min IV) or TCI (3–6 ng/ml) or alfentanil (0.2–0.5 ug/kg/min IV) Desflurane or sevoflurane: maintaining 1 MAC

Anesthesia during CPB	Anesthesia during CPB
Morphine (10 mg IV bolus) start of CPB	Propofol 1–2 mg/kg/h maintaining a Bispectral Index of 40–60
Volatile anesthesia maintaining a Bispectral Index of 40–60	Continue intraoperative analgesia infusions

Post CPB	Post CPB
No reversal agent administered	Sugammadex for reversal (200 mg IV)
Morphine/oxycodone (5–10 mg IV) at discretion of the treating anesthesiologist	Additional analgesia with small aliquots fentanyl (50–100 ug) or morphine/oxycodone (5–10 mg IV)
Nasogastric or oral gastric tube left in situ to decompress stomach and suction any gastric contents	Single pass oral gastric tube to decompress stomach and suction any gastric contents, then removed
No paracetamol or ketamine	Stop remifentanil or alfentanil during insertion of sternal wires Start ketamine 0.1 mg/kg/h Paracetamol 1 g IV

Extubation	Extubation
Transfer from operating room to to ICU Endotracheal tube in-situ Mechanically ventilated and sedated At discretion of attending intensive care clinician: • Weaning from mechanical ventilation • Spontaneous breathing trial • Enodotracheal tube extubation • Transfer to cardiac care unit ward bed	At completion of surgery stop anesthesia agents and initiate spontaneous breathing trial: • Continuous reduction in continuous positive airway pressure to minimal ventilator settings i.e. 5 cmH_2_0 pressure support and 2–5 mmH_2_O PEEP (ensure arterial saturation > 92%, PaCO_2_ < 55 mmHg, FiO_2_ < 0.4, PaO_2_/FiO_2_ > 200 and respiratory rate < 20 b/min; • Adequate mentation • Pain controlled • Head lift off pillow, raise arms in air for 10 s • Stable hemodynamics • Normothermia Extubate on operating table or transfer patient on propofol infusion (20–40 mg/h) to ICU for continuation of spontaneous breathing trial and extubation in ICU

Postoperative analgesia	Postoperative analgesia
Opioid patient controlled analgesia (PCA) at discretion of treating intensivist. Type of opiod and opioid regime not standardized	Patient controlled analgesia (PCA) fentanyl (10 ug/bolus, 5 min boluses, 5 min lockout, no background infusion)
Paracetamol (1 g IVI) at discretion of treating intensivist	Paracetamol (1 g IVI) every 6 h for 48 h
Adjunctive analgesia per ICU	Tramadol (50–100 mg IV) or ketamine (0.05–0.1 mg/kg/h IV) if refractory analgesia
No acute pain service review	Acute pain service review twice daily

Postoperative agitation/delirium	Postoperative agitation/delirium
Sedation/antipsychotics at discretion of treating intensivist	Dexmedetomidine (0.2 ug/kg/h)

This table highlights the important differences between patients receiving the fast-track protocol compared to standard protocol. Notable differences occur throughout the care process, with more specific guidelines being introduced for postoperative care in the FTCA group including extubation, analgesia and management of delirium

**Fig. 1:**
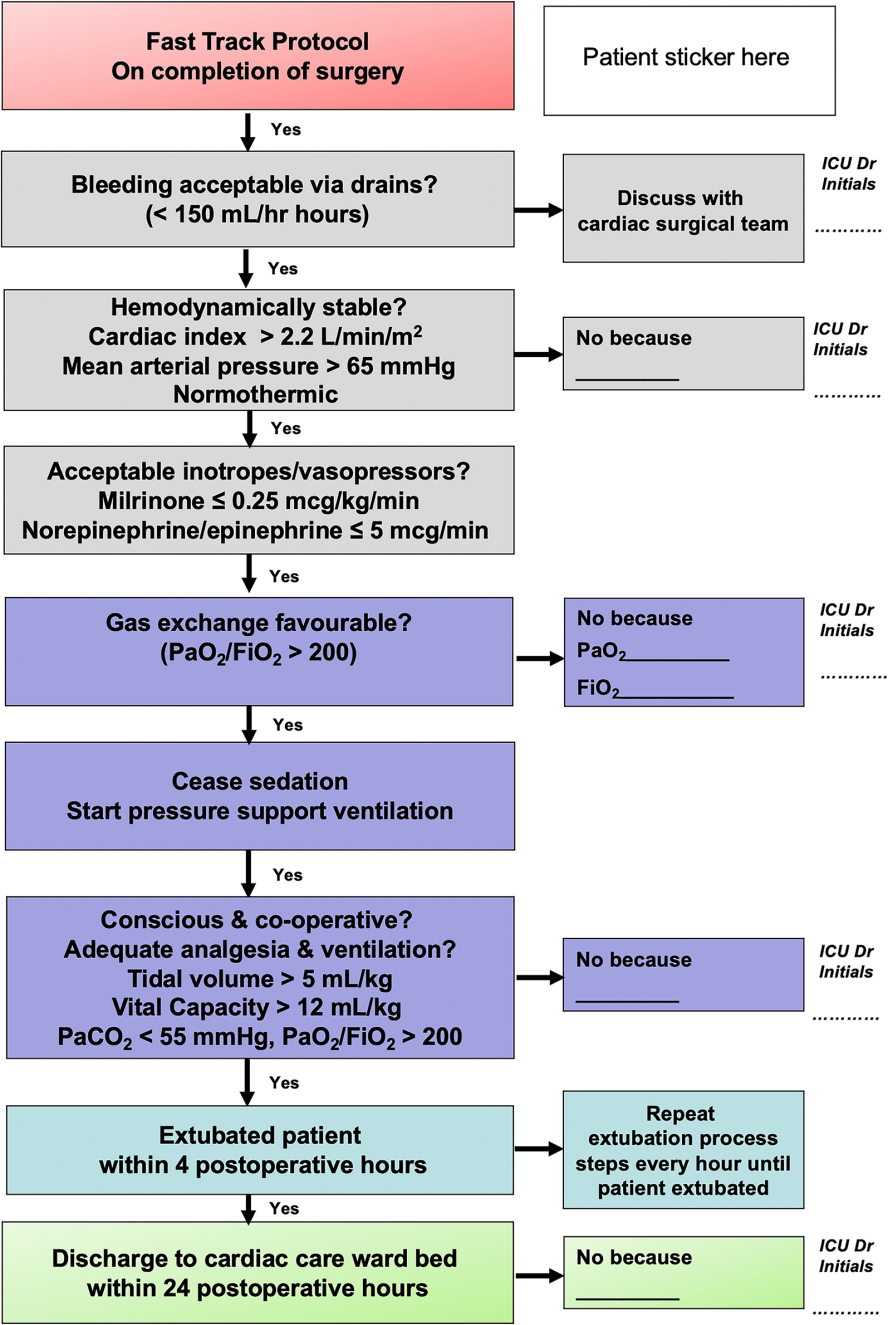
Fast-track cardiac protocol at completion of surgery. *PaO*_*2*_ partial pressure of oxygen in arterial blood, *FiO*_*2*_ fraction of inspired oxygen, *PaCO*_*2*_ partial pressure of carbon dioxide in arterial blood

We also developed a dedicated postoperative protocol (Fig. 1) specifically to standardize the decision to allow the patient to emerge from general anesthesia immediately after surgery and remove the endotracheal tube. Assessable metrics were selected through multi-disciplinary collaboration between anesthesiologists, intensivists and cardiac surgeons, and included assessment of postoperative bleeding, stability of hemodynamic parameters, vasopressor requirement and adequacy of gas-exchange. If these criteria were met, sedation was weaned and pressure support ventilation was attempted. If weaning was successful and the patient was conscious and co-operative, each FTCA patient was extubated within 4 h post-operatively.

Our defined FTCA program included all adult patients receiving elective, primary cardiac bypass graft or valve surgery requiring on-pump cardiopulmonary bypass. We excluded all patients having emergency surgery, redo-surgery, off pump coronary artery bypass surgery, preoperative poor left ventricular function (ejective fraction < 40%), moderate pulmonary hypertension (pulmonary artery systolic pressure > 50 mmHg), underlying respiratory disease (FEV1 < 75% predicted), or any other complex cardiac surgery (defined as double valve surgery, ascending, arch or descending aortic surgery, surgery for congenital heart disease). Appropriate FTCA candidates were identified in surgical and anesthesia pre-admission clinic and enrolled between October 2015 and Jan 2016.

Patient characteristics including demographics, weight, indication for surgery, preoperative comorbidities and Euroscore were recorded_,_ as were aortic cross clamp and cardiopulmonary bypass times [8]. We collected the rates of adherence to the prescribed FTCA protocols, time until extubation and discharge to the ward, and reasons for FTCA failure. We recorded the incidence of postoperative complications, ventilation time, ICU and hospital length of stay and 30-day mortality.

Descriptive statistics were generated for the data collected using GraphPad Prism version 8.3.0 (GraphPad Software, La Jolla, CA, USA) and graphical representations of the data were generated using Microsoft Excel version 1910 for windows (2016, Microsoft Corp., WA, USA). Data are presented as mean [standard deviation (SD)], median [interquartile range (IQR)] and number (percentile).

### Results

Of the 16 patients enrolled in the FTCA group, 12 (75%) were male. The median (IQR, min–max) age was 56 years (40:66; 36–81). Mean (SD) body mass index was 29.3 (4.6) and Euroscore was 1.5 (0.9:2.2), respectively. Three patients (19%) had controlled diabetes, 8 patients (50%) had controlled hypertension, and 12 patients (75%) hypercholesterolemia. No patients had a history of smoking, peripheral vascular disease or chronic obstructive pulmonary disease.

**Fig. 2:**
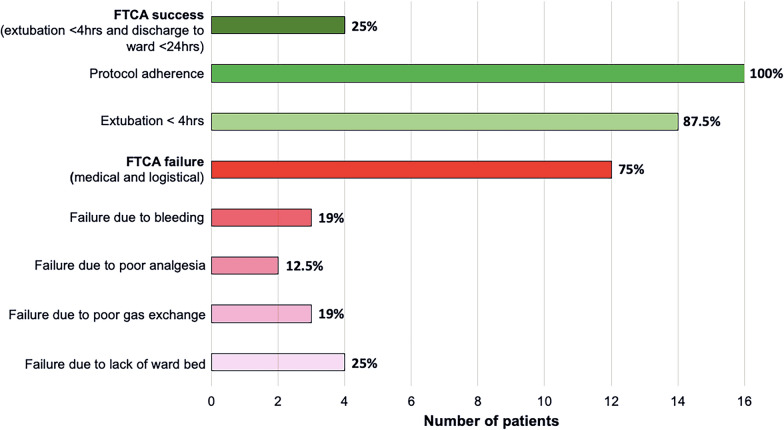
Fast-track cardiac anesthesia (FTCA) feasibility

We observed 100% compliance with the intraoperative and postoperative protocols with no protocol violations. The program success rate was 25%. Reasons for FTCA failure included logistical factors: ward bed was unavailable for 4 patients (25%) who were ready for discharge from the ICU, and patient factors, which included poorly controlled postoperative analgesia [2 patients (12.5%)], poor gas exchange [3 patients (19%)], and postoperative bleeding [3 patients (19%)]. Of the 16 patients, 5 (31%) were extubated in the operating room on-table, 9 (56%) were extubated within 4 h of ariving in the ICU, and 2 (12.5%) were extubated within 18 h (Fig. 2).

The mean (SD) cardiopulmonary and aortic clamp times were 101.4 (45.0) and 81.4 (44.8) min, respectively. The median (IQR, min–max) duration of postoperative ventilation was 2.1 (0:6.5; 0–34) h. Four patients were successfully extubated and discharged from ICU within 24-h of surgery. Intravenous ketamine infusion (0.05–0.1 mg/kg/h) was administered to 3 patients in ICU. There were no adverse effects observed from the ketamine infusiuon and no patient required treatment for postoperative delirium or cognitive dysfunction. The median (IQR, min–max) duration of ICU stay was 22 (12.2:40.5, 4–94) h. Total median (IQR, min–max) duration for hospital length of stay was 6 (5.2:8.5; 4–16) days. There were no readmissions to ICU or postoperative mortality.

### Discussion

We developed a FTCA program and assessed the program’s feasibility and compliance in a small cohort of patients undergoing cardiac surgery. Reasons why patients did not complete the FTCA pathway included ongoing need for mechanical ventilation, high analgesic requirements and lack of cardiac ward beds. These factors are current barriers to the further development and expansion of this program at our institution; however, they are all potentially modifiable.

Our study provides some novel findings. Firstly, that pain and post-extubation pulmonary factors often impact on FTCA success. Secondly, despite dedicated funding to facilitate a postoperative cardiac ward bed, 50% of enrolled patients failed FTCA due to lack of a ward bed. To our knowledge, neither post-extubation pain, nor oxygenation have been previously described as inhibiting FTCA success and there are no published reports describing lack of post-ICU beds as a cause of FTCA failure. It has previously been reported that FTCA protocols perform better in dedicated units, with a prospective 2014 study concluding cardiac-care was superior to general ICU care for facilitating FTCA demonstrating considerably shorter length of stay [9].

Our findings are consistent with earlier studies, including a 2015 randomized controlled trial, suggesting that FTCA results in shorter ICU length of stay without increase in postoperative complications in low to moderate risk patients [10]. However, persistent concerns regarding risk of adverse events and patient safety remain a major factor limiting the implementation of widespread FTCA protocols. The available evidence suggests otherwise, with a 2013 retrospective study evaluating safety of FTCA analysing data from 7000 patients and concluded there is no evidence of an increased risk of adverse outcomes in patients undergoing FTCA [11]. Likewise, the two most recent Cochrane reviews into FTCA safety concluded that fast-track interventions have similar risks compared to standard care, and that FTCA protocols are safe in low to medium risk patients [4, 12]. The safety of FTCA and equivalence in outcomes suggests the potential to expand this approach to also consider nurse-lead FTCA protocols as potentially safe and effective [13]. When used in conjunction with advancements in surgical techniques such as minimally invasive incisions, robot-assisted cardiac surgery and rapid-deployment valves there are likely to be a range of opportunities to further improve resource utilization, reduce postoperative complications and expedite recovery [14, 15].

Our study used Euroscore calculation to assess perioperative risk, using widely accepted definitions of low-moderate cardiac risk (a Euroscore less than or equal to 5) to select patients appropriate for FTCA [16]. This definition is imperfect, however, and more work is required to optimize predictive systems, especially given the development of the updated Euroscore II system, which may also overestimate the risk of mortality in lower risk patients and underestimate the risk of mortality in higher risk patients [17, 18].

Preoperative risk calculators such as the Euroscore, may help predict FTCA failure, with evidence suggesting American Society of Anesthesiologists (ASA) class and New York Heart Association (NHYA) class, as well as total operation time are strong predictors of fast-track failure [19]. A 2013 retrospective observational study of 229 patients determined ASA physical classification over 3, NHYA classification over 3 and total operation time (over 267 min) are strong, independent predictors of FTCA failure [19]. Conversely, a 2013 retrospective analysis of over 11,000 patients found age and left ventricular dysfunction were significant preoperative predictors of failure FTCA [20]. Most recently, a 2018 prospective observational study concluded that age, pre-existing renal impairment, Euroscore, inotropic requirement, aortic cross-clamp time and total cardiopulmonary bypass time were all predictors of FTCA failure [21]. To summarize, the available evidence suggests that, predictably, FTCA is more likely to be successful in younger patients with fewer comorbidities and greater baseline cardiac function, with ongoing work required to determine hard criteria for FTCA patient selection.

Our work has helped identify key challenges to overcome if successful fast-track programs are to be implemented. With 12.5% of patients failing to be discharged from ICU secondary to concerns regarding analgesia, our study identified pain management as an important area to address for FTCA success. Evidence has identified that specialist post-operative analgesia regimes are required, with further evidence suggesting opioid PCAs are superior to nurse-initiated analgesia for this patient group [9, 22]. With 25% of patients failing due to the lack of an appropriate cardiac step-down bed, despite specific institutional funding and approval for this project flags bed access as a major issue obstructing FTCA success. A potential challenge associated with ensuring smooth, efficient transition from ICU to ward-based are comes with the large variations in what defines an adequate cardiac “step-down” bed, and the associated variations in delivery of care [23].

### Conclusion

FTCA programs offer potentially more efficient use of healthcare resource and improved access for patients. After trialing a dedicated FTCA program as part of a quality improvement initiative, we observed that the implementation of a fast-track program was feasible, potentially beneficial in reducing ventilation and ICU times, and not associated with an increase in complications or mortality. Major limitations to implementing a FTCA program include the availability of appropriate step-down cardiac ward beds, as well as challenges relating to respiratory status and pain management.

## Limitations

We acknowledge several limitations of our study. This is a single center study, with findings being potentially influenced by local institutional policies and practices. Comparing existing audit data, our study group were younger and less unwell than that of many elective cardiac surgery cohorts, further limiting such comparisons. We enrolled low patient numbers, being not sufficiently powered to definitively compare complication rates, however this was neither an interventional, nor prospective observational study. Finally, we did not evaluate any cost benefits of the program, however this was not the purpose of our study, and has been well documented by others [24].

## Data Availability

All data produced or analysed during the current study are included in this article. The original dataset is available upon request to the corresponding author.

## Abbreviations

FTCA: Fast-track cardiac anesthesia
AUD: Australian Dollars
ICU: Intensive care unit
PDSA: Plan-do-study-act
USA: United States of America
CA: California
WA: Washington
SD: Standard deviation
IQR: Interquartile range
ASA: American Society of Anesthesiologists
NYHA: New York Heart Association
PCA: Patient-controlled analgesia

## References

[1] Health AIo (2019). Cardiovascular disease.

[2] Health AIo (2019). Elective surgery waiting times 2017–18.

[3] Design, service and infrastructure plan for Victoria’s cardiac system. In: Department of Health and Human Services SoV editor. Melbourne: Victorian Government; 2016.

[4] Wong WT, Lai VK, Chee YE, Lee A (2016). Fast-track cardiac care for adult cardiac surgical patients. Cochrane Database Syst Rev.

[5] Bainbridge D, Cheng D (2017). Current evidence on fast track cardiac recovery management. Eur Heart J Suppl.

[6] Warltier DC, Myles PS, Daly DJ, Djaiani G, Lee A, Cheng DCH (2003). A systematic review of the safety and effectiveness of fast-track cardiac anesthesia. Anesthesiol J Am Soc Anesthesiol.

[7] Taylor MJ, McNicholas C, Nicolay C, Darzi A, Bell D, Reed JE (2014). Systematic review of the application of the plan-do-study-act method to improve quality in healthcare. BMJ Qual Saf.

[8] Cove ME, Ying C, Taculod JM (2016). Multidisciplinary extubation protocol in cardiac surgical patients reduces ventilation time and length of stay in the intensive care unit. Ann Thorac Surg.

[9] Probst S, Cech C, Haentschel D, Scholz M, Ender J (2014). A specialized post-anaesthetic care unit improves fast-track management in cardiac surgery: a prospective randomized trial. Crit Care.

[10] Salah M, Hosny H, Salah M, Saad H (2015). Impact of immediate versus delayed tracheal extubation on length of ICU stay of cardiac surgical patients, a randomized trial. Heart Lung Vessels.

[11] Svircevic V, Nierich AP, Moons KG, Brandon Bravo Bruinsma GJ, Kalkman CJ, van Dijk D (2009). Fast-track anesthesia and cardiac surgery: a retrospective cohort study of 7989 patients. Anesth Analgesia.

[12] Zhu F, Lee A, Chee YE (2012). Fast-track cardiac care for adult cardiac surgical patients. Cochrane Database Syst Rev.

[13] Serena G, Corredor C, Fletcher N, Sanfilippo F (2019). Implementation of a nurse-led protocol for early extubation after cardiac surgery: a pilot study. World J Crit Care Med.

[14] Di Eusanio M, Vessella W, Carozza R (2018). Ultra fast-track minimally invasive aortic valve replacement: going beyond reduced incisions. Eur J Cardio-Thorac Surg.

[15] Tarola CL, Al-Amodi HA, Balasubramanian S (2017). Ultrafast track robotic-assisted minimally invasive coronary artery surgical revascularization. Innovations (Philadelphia, Pa).

[16] Nashef SA, Roques F, Michel P, Gauducheau E, Lemeshow S, Salamon R (1999). European system for cardiac operative risk evaluation (EuroSCORE). Eur J Cardio-Thorac Surg.

[17] Nashef SAM, Roques F, Sharples LD (2012). EuroSCORE II†. Eur J Cardiothorac Surg.

[18] Guida P, Mastro F, Scrascia G, Whitlock R, Paparella D (2014). Performance of the European system for cardiac operative risk evaluation II: a meta-analysis of 22 studies involving 145,592 cardiac surgery procedures. J Thorac Cardiovasc Surg.

[19] Kiessling AH, Huneke P, Reyher C, Bingold T, Zierer A, Moritz A (2013). Risk factor analysis for fast track protocol failure. J Cardiothorac Surg.

[20] Haanschoten MC, van Straten AHM, ter Woorst JF (2012). Fast-track practice in cardiac surgery: results and predictors of outcome. Interact Cardiovasc Thorac Surg.

[21] Tham YC, Tan Z, Tam ALW (2015). Improving on fast-track protocol for post cardiac surgery patients. J Cardiothorac Surg.

[22] Zubrzycki M, Liebold A, Skrabal C (2018). Assessment and pathophysiology of pain in cardiac surgery. J Pain Res.

[23] Prin M, Wunsch H (2014). The role of stepdown beds in hospital care. Am J Respir Crit Care Med.

[24] Cheng DC, Wall C, Djaiani G (2003). Randomized assessment of resource use in fast-track cardiac surgery 1-year after hospital discharge. Anesthesiology.

